# Leishmaniasis in Refugee and Local Pakistani Populations

**DOI:** 10.3201/eid1009.040179

**Published:** 2004-09

**Authors:** Simon Brooker, Nasir Mohammed, Khaksar Adil, Said Agha, Richard Reithinger, Mark Rowland, Iftikhar Ali, Jan Kolaczinski

**Affiliations:** * London School of Hygiene and Tropical Medicine, London, United Kingdom;; † HealthNet International, Peshawar, Pakistan

**Keywords:** anthroponotic cutaneous leishmaniasis, epidemiology, geographical information systems, Pakistan, Afghan refugees, control, dispatch

## Abstract

The epidemiology of anthroponotic cutaneous leishmaniasis was investigated in northwest Pakistan. Results suggested similar patterns of endemicity in both Afghan refugee and Pakistani populations and highlighted risk factors and household clustering of disease.

In Central Asia, anthroponotic cutaneous leishmaniasis (ACL) is commonly caused by *Leishmania tropica* and characterized by large, chronic, and disfiguring skin ulcers, which often cause severe social stigma. Because ACL is transmitted anthroponotically (i.e., from human to human) by sandflies, the infection can spread rapidly in concentrated populations, particularly under poor housing conditions, i.e., overcrowding or lack of protection from bloodsucking insects. In Afghanistan, the incidence of endemic but sporadic ACL has dramatically increased during decades of civil war, because of the associated deterioration of the infrastructure and migration (*1**–**3*). Less is known about the current distribution of the disease in neighboring Pakistan, where it has always been widespread but considered "patchy" and nonendemic (*4*). Recently, however, local authorities and nongovernmental health providers have reported an increasing number of ACL cases in Afghan refugee camps (*5**,**6*), which causes concern about the potential spread of the disease among the population and local Pakistani villagers. Therefore, a large-scale epidemiologic study was conducted throughout northwest Pakistan to investigate this issue.

## The Study

From December 2002 to March 2003, a study was conducted in 48 Afghan refugee camps and 19 neighboring villages in Balochistan and North-West Frontier Province (NWFP), Pakistan. Refugee camps were selected on the basis of past and present ACL cases reported by healthcare providers. Villages within 1 km of selected camps were included in the survey; if multiple villages were within 1 km of a camp, one with reported ACL cases was randomly selected, although this method may have introduced selection bias. The goal of the study was to estimate the prevalence of ACL in Afghan refugee camps and neighboring Pakistani villages, as well as determine whether refugee camps could be the source of the anecdotal rise in ACL cases in neighboring villages. In each site, 40 households were sampled along east-west and north-south perpendicular transects. Every head of household was interviewed with a standard questionnaire. If a family reported cases of ACL, an interviewer who had been trained in clinical ACL diagnosis asked to inspect the lesions. Because of logistic constraints, no parasitologic confirmation was performed, but lesions caused by organisms other than *Leishmania* are rare, and our previous studies have shown that specificity of our clinical diagnosis is 73%–76% (*5*).

The study included 21,046 persons in 48 refugee camps and 7,305 persons in 19 neighboring villages. Overall, 650 persons (2.3%) had ACL lesions only, 1,236 (4.4%) had ACL scars only, and 38 persons had both ACL lesions and scars. Of those with active ACL, the mean lesion number was 2.1 (range 1–16), and the mean lesion duration (to survey date) was 5.1 months (range 0.7–50 months). Using maximum likelihood methods (*7*), we estimated the average annual force of infection of ACL to be 0.01 per year (10 cases/1,000 persons per year) during the past 6 years.

In refugee camps, the prevalence of ACL lesions was 2.7%, and prevalence of scars was 4.2%. In neighboring Pakistani villages, the prevalence of ACL lesions was 1.7%, and prevalence of scars was 4.9%. Lesion prevalence increased with age more markedly among local Pakistanis than Afghan refugees until children were 5–6 years of age; then the prevalence of lesions decreased among Pakistanis and was lower than in the Afghan refugee population for all remaining age groups (Figure). These age trends suggest past infection and resultant immunity. Had the disease been introduced more recently, the risk of ACL would not be expected to be related to age, since everyone would be susceptible to infection (*8*). However, the low prevalence of scars relative to the number of active lesions, especially among adults, suggests that the disease has been endemic in the region for a short period of time and that transmission may be characterized by a prolonged epidemic similar to that found in Kabul (*2**,**4*).

**Figure:**
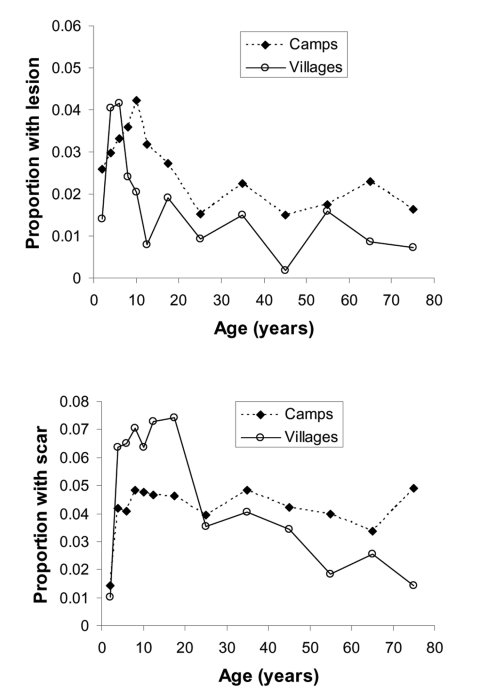
A) Proportion of unscarred population with active lesions by age and settlement type. B) Proportion of population with scar by age and settlement type.

To examine the association with potential risk factors and to take clustering of persons within households into account, univariate odds ratios (OR) were estimated by logistic regression with robust standard errors. We used backward stepwise multiple logistic regression to identify significant explanatory risk factors while controlling for other variables. Spatial clustering of ACL was investigated at the household and village levels. The degree of within-household clustering was calculated by using a random-effects model fitted to a logistic regression to account for the nonindependence of persons within households. The analysis was conducted using STATA 8 (Stata Corporation, College Station, TX). The nonparametric Mantel correlation statistic with Mantel 2 (Queensland University of Technology, Brisbane, Australia) was used to assess spatial correlation in prevalence between settlements by investigating the relationship between differences in lesion prevalence and geographic distances.

The univariate analysis showed that an increased risk of ACL lesion was associated with years lived in camp or village, a family member visiting Afghanistan in the last 12 months, household members with ACL lesions, household members having ACL scars, age group, household with stone walls, crowding in the household (i.e., the number of people per room), having cows in a compound, and having dogs in a compound (Table 1). The same variables were significantly associated with the risk of having an ACL scar, with the exception of a family member's having visited Afghanistan in last 12 months. Use of a mosquito net was associated with an increased risk of having a scar. Multivariate analysis showed that younger age, as well as ACL lesions in other household members, increased the risk of an ACL lesion (Table 2). Increased risk of an ACL scar was associated with younger age, living in a refugee camp, and scars in other household members. No significant interactions were detected among the other variables included in the analysis. Finally, after age, sex, and household factors were adjusted for, the random effects model found evidence for significant household clustering of active ACL cases: ρ = 0.54 (95% confidence interval [CI] 0.49–0.59, p < 0.0001). ACL scars clustered in households to an even greater degree: ρ = 0.62 (95% CI 0.59–0.65, p < 0.05). The prevalence of ACL lesions showed a marked variation (0%–21.9%) between Afghan refugee camps and neighboring Pakistani villages. However, analysis using the Mantel correlogram indicated no spatial structuring of ACL between neighboring villages, which emphasizes the highly focal distribution of ACL transmission at the village level and corroborating significant household clustering of ACL.

**Table 1:** Unadjusted odds ratios for variables associated with the risk of anthroponotic cutaneous leishmaniasis lesion and scar^a^

Variable	Lesion [OR (95% CI)]	Scar [OR (95% CI)]
Village^b^	χ^2^ = 540, df = 66, p < 0.001	χ^2^ = 786, df = 66, p < 0.001
Refugee camp (compared to local village)	1.540 (1.16–2.06), p = 0.003	0.82 (0.62–1.09), p = 19
Nationality (Afghan compared to Pakistani)	1.050 (0.78–1.38), p = 0.720	0.940 (0.70–1.26), p = 0.680
Years lived in camp/village	1.010 (1.01–1.02), p < 0.001	1.002 (0.99–1.01), p = 0.510
Family member visited Afghanistan in last 12 mo.	1.740 (1.37–2.20), p < 0.001	1.690 (1.35–2.11), p < 0.001
Lesion prevalence in other household members	1.120 (1.11–1.13), p < 0.001	1.040 (1.03–1.06), p < 0.001
Scar prevalence in other household members	1.030 (1.02–1.03), p < 0.001	1.090 (1.08–1.10), p < 0.001
Sex (female compared to male)	1.010 (0.89–1.15), p = 0.770	1.050 (0.95–1.16), p = 0.310
Age group (compared to 0–4 y)		
5–19 y	1.090 (0.87–1.37), p = 0.450	1.750 (1.44–2.13), p < 0.001
>20 y	0.560 (0.43–0.71), p < 0.001	1.080 (1.07–1.59), p = 0.007
Type of wall (compared to mud)		
Brick	0.940 (0.33–2.68), p = 0.920	0.640 (0.31–1.31), p = 0.220
Stone	0.530 (0.32–0.88), p = 0.010	0.480 (0.30–0.77), p = 0.002
Other	2.000 (0.76–5.21), p = 0.150	1.160 (0.59–2.31), p = 0.650
Type of ceiling (compared to cloth)		
Concrete	0.690 (0.26–1.79), p = 0.450	1.090 (0.44–2.71), p = 0.850
Wood (beam)	1.430 (0.65–3.17), p = 0.370	1.590 (0.73–3.48), p = 0.240
Wood (thatched)	0.460 (0.16–1.27), p = 0.140	1.770 (0.70–4.49), p = 0.230
Other	0.850 (0.30–2.41), p = 0.760	1.180 (0.47–2.99), p = 0.710
Rooms/person	0.200 (0.07–0.56), p = 0.002	0.430 (0.21–0.91), p = 0.020
Cows in compound (yes/no)	1.420 (1.22–1.65), p < 0.001	1.510 (1.34–1.69), p < 0.001
Dogs (yes/no)	1.660 (1.42–1.94), p < 0.001	1.310 (1.16–1.48), p < 0.001
Meshed windows (% windows covered)	1.260 (0.51–3.13), p = 0.610	0.720 (0.32–1.61), p = 0.420
Use mosquito net	1.180 (0.83–1.67), p = 0.350	1.560 (1.19–2.05), p = 0.001
Treated mosquito net	0.760 (0.32–1.78), p = 0.530	0.740 (0.42–1.32), p = 0.320

^a^OR, odds ratio; CI, confidence interval; df, degrees of freedom. ^b^The overall significance of this categorical variable is shown rather than the 67 different survey locations.

**Table 2:** Adjusted odds ratios associated with risk of anthroponotic cutaneous leishmaniasis lesion and scar, based on multiple logistic regression model, using village as a random effect variable

Variable	Adjusted OR (95% CI)^a^
**Lesion**	
Age group (compared to 0–4 y)	
5–19 y	1.17 (0.90–1.52) p = 0.320
>20 y	0.48 (0.35–0.65) p < 0.001
Lesion prevalence in other household members	1.10 (1.09–1.11) p < 0.001
**Scar**	
Age group (compared to 0–4 y)	
5–19 y	2.52 (1.93–3.29) p < 0.001
>20 y	1.99 (1.48–2.69) p < 0.001
Refugee camp (compared to local village)	1.48 (1.03–2.14) p = 0.040
Scar prevalence in other household members	1.08 (1.07–1.11) p < 0.001

^a^OR, odds ratio; CI, confidence interval.

## Conclusion

The analysis of putative risk factors for ACL indicated that living in a stone house reduced the risk, whereas the presence of cows and dogs increased it (Table 2). Although dogs have been found infected with *L. tropica* (*9*), they are probably not leishmaniasis reservoirs, as transmission of *L. tropica* is thought to be anthroponotic (*2*). Instead, dogs and other domestic animals represent an additional feeding source for sandflies, which increases contact between vectors and humans. Improved housing protects against vector-borne diseases, since it reduces human-vector exposure. Reported household use of a mosquito net was associated with increased risk of ACL scar, which may reflect the practice of selling insecticide-treated nets at highly subsidized prices to refugee households with active ACL.

Although parasite identification was not carried out in this study, that *L. tropica* is the etiologic agent seems probable because it causes most leishmaniasis cases in Central Asia (*5**,**10*), and transmission is characterized by clustering of cases and higher risk among children. Our data indicate that parasite transmission is autochthonous in surveyed sites, although highly heterogeneous between sites. Observed childhood-acquired immunity indicates that not all cases are imported from Afghanistan, as has been suggested (*5*). Consequently, continual and vigilant surveillance is required to monitor the epidemiology of ACL in the region. The mass return of *Leishmania-*infected refugees to urban areas in Afghanistan poses a particular risk, since housing is often poor, and living conditions are crowded. Including ACL prevention measures in Afghanistan's basic package of health services (e.g., supplying insecticide-treated nets to areas at high risk) should be considered to prevent the spread of disease through previously ACL-free urban areas.

Current ACL interventions in the study areas in Pakistan are funded by the United Nations High Commissioner for Refugees (UNHCR) and mainly focus on Afghan refugees. Free diagnosis on the basis of clinical symptoms, analysis of specimens by microscope, and treatment with antimony are provided for all patients attending basic health units in refugee camps, and insecticide-treated nets are sold at highly subsidized prices to refugees with active ACL. The local population is not a focus of the program, since resources are limited. Insecticide-treated net users in local villages either make their own nets or acquire them through "leakage" of nets intended for Afghan refugees or at communities across the border in Afghanistan. Long-term control of ACL transmission in Pakistan will require extending diagnostic and treatment services and building up a program to sell insecticide-treated nets to the local population. With the ongoing reduction in UNHCR funding and anticipated phasing-out of support to refugee health care at the end of 2005, the population will depend on the Pakistan Ministry of Health to deliver these much needed services.
